# T-helper cells and their cytokines in pathogenesis and treatment of asthma

**DOI:** 10.3389/fimmu.2023.1149203

**Published:** 2023-06-12

**Authors:** Tingfen Ji, Hequan Li

**Affiliations:** Department of Respiratory and Critical Care Medicine, the First Affiliated Hospital, School of Medicine, Zhejiang University, Hangzhou, Zhejiang, China

**Keywords:** bronchial asthma, cell, Th2, cell, Th17, helper T cells, molecular targeted therapy

## Abstract

Prosperous advances in understanding the cellular and molecular mechanisms of chronic inflammation and airway remodeling in asthma have been made over the past several decades. Asthma is a chronic inflammatory disease of the airways characterized by reversible airway obstruction that is self-resolving or remits with treatment. Around half of asthma patients are “Type-2-high” asthma with overexpression of type 2 inflammatory pathways and elevated type 2 cytokines. When stimulated by allergens, airway epithelial cells secrete IL-25, IL-33, and TSLP to derive a Th2 immune response. First ILC2 followed by Th2 cells produces a series of cytokines such as IL-4, IL-5, and IL-13. T_FH_ cells control IgE synthesis by secreting IL-4 to allergen-specific B cells. IL-5 promotes eosinophil inflammation, while IL-13 and IL-4 are involved in goblet cell metaplasia and bronchial hyperresponsiveness. Currently, “Type-2 low” asthma is defined as asthma with low levels of T2 biomarkers due to the lack of reliable biomarkers, which is associated with other Th cells. Th1 and Th17 are capable of producing cytokines that recruit neutrophils, such as IFN-γ and IL-17, to participate in the development of “Type-2-low” asthma. Precision medicine targeting Th cells and related cytokines is essential in the management of asthma aiming at the more appropriate patient selection and better treatment response. In this review, we sort out the pathogenesis of Th cells in asthma and summarize the therapeutic approaches involved as well as potential research directions.

## 1 Introduction

**Figure 1:**
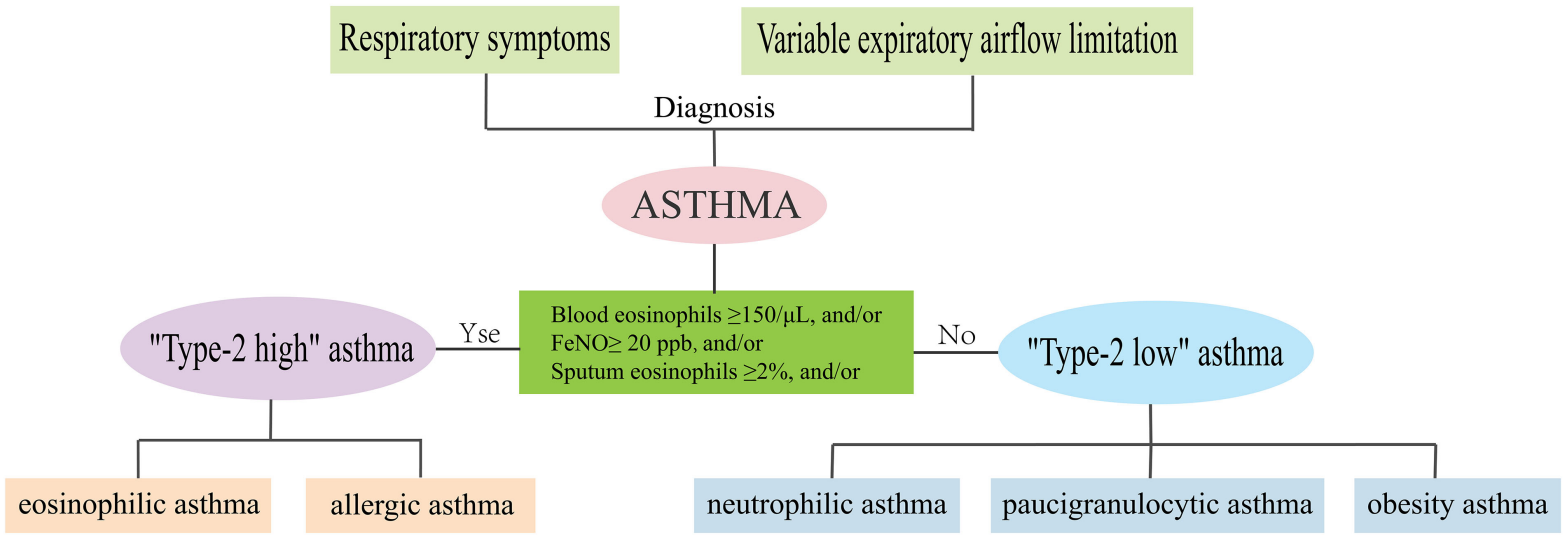
Phenotypes and endotypes of asthma.

Asthma is a heterogeneous disease, characterized by major differences in patients’ clinical manifestations, severity, response to treatment, and allergens stimulation (1). With intensive research on the underlying molecular mechanisms or endotypes of asthma, in recent years, the asthma population has been divided into “Type-2-high” asthma and “Type-2-low” asthma (2). “Type-2-high” asthma is characterized by high secretion of interleukin (IL)-4, IL-5, and IL-13, elevated eosinophil counts, serum periostin, and total IgE, including allergic and eosinophilic asthma (3, 4). “Type-2-low” asthma occurs in the absence of T2 biomarkers or eosinophilic elevation, including neutrophilic, paucigranulocytic, and obesity asthma (4, 5) (**Figure 1**).

The two endotypes of asthma involve different Th cell-driven inflammatory responses and airway remodeling processes, including Th2, T follicular helper (T_FH_), Th17, and Th1 cells. Activation of Th2-pathways is at the core of type 2 inflammation, producing excessive amounts of the cytokines IL-4, IL-5, and IL-13. IL-5 induce eosinophil activation, maturation, and recruitment, while IL-4 and IL-13 are involved in goblet cell metaplasia, airway smooth muscle(ASM) contractility, and airway hyperresponsiveness(AHR) (6). T_FH_ cells regulate B cell proliferation and conversion of immunoglobulin classes, particularly in germinal center structures (7). These cells produce IL-21 and IL-4, which are essential for B-cell stimulation and play a key role in controlling IgE production in asthma (8, 9). Accumulating evidence suggests that “Type-2-low” asthma is associated with abnormal Th17 or Th1 cell immune responses. Th17 cells producing the IL-17 family are involved in neutrophil inflammation and airway remodeling processes and are responsible for corticosteroid resistance in asthma (10). Th1 cell activation promoting interferon (IFN)-γ and tumor necrosis factor (TNF)-α production also contributes to driving neutrophil inflammation (11). Some other Th cell subsets such as Th9 and Th22 have been described by some laboratories as being involved in the secretion of IL-9 and IL-22, respectively, which are associated with airway inflammation and AHR in asthma (12, 13). However, defining them as distinct subgroups remains controversial. It is unclear if these subsets are stable or are indeed relevant to asthma pathogenesis outside animal models.

**Figure 2:**
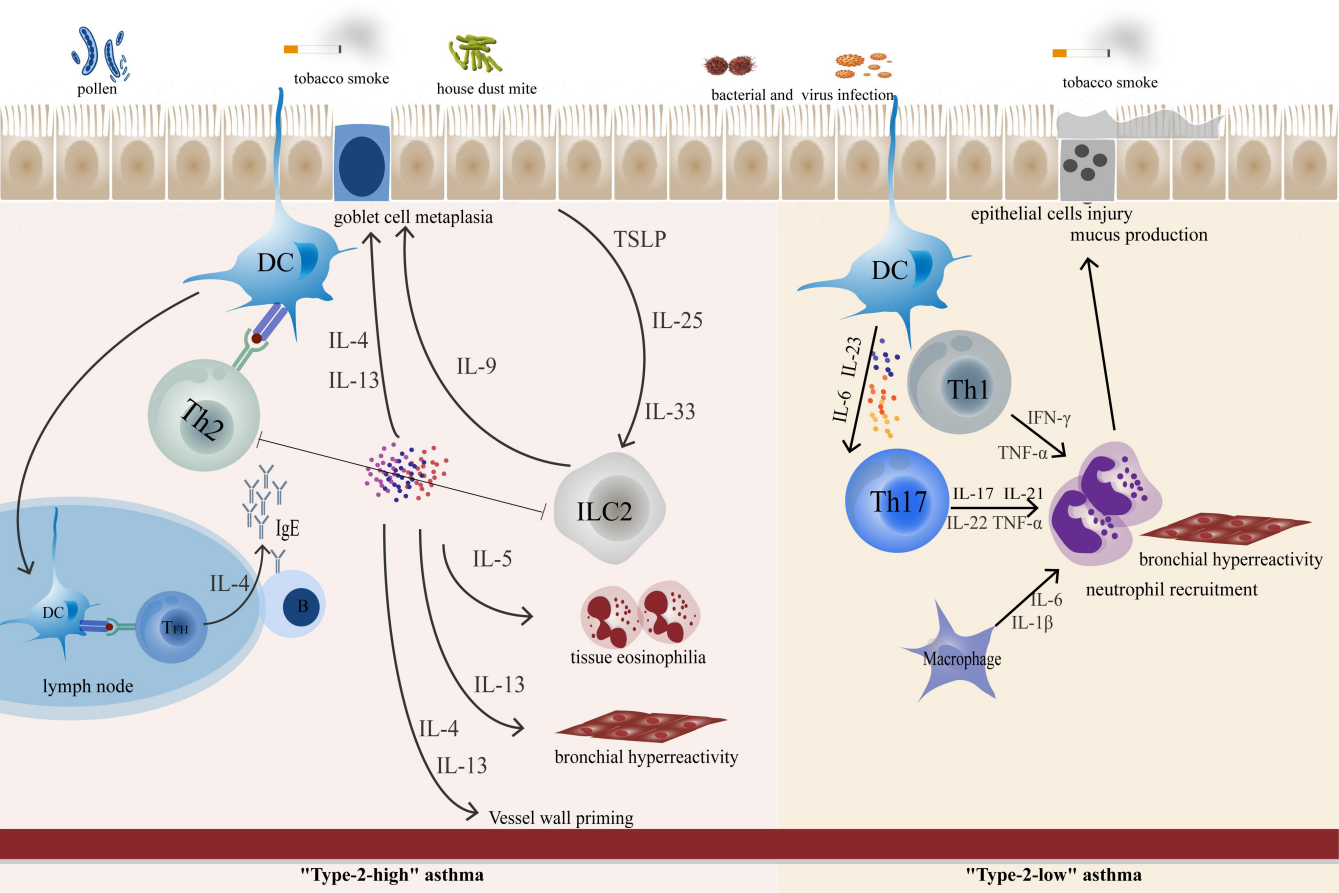
In “Type-2 high” asthma, epithelial-derived TLPS, IL-25, and IL-33 can awaken ILC2. Cytokines produced by epithelial cells promote DC function, polarize CD4^+^ T cells and promote the polarization of Th2 cells. T_FH_ cells control IgE synthesis by secreting IL-4 to allergen-specific B cells. ILC-derived IL-9, which promotes goblet cell metaplasia. IL-5 promotes eosinophil inflammation, while IL-13 and IL-4 are involved in goblet cell metaplasia and bronchial hyperresponsiveness. In “Type-2-low” asthma, Th17 and Th1 play an important role in airway neutrophil inflammation and airway remodeling.

Currently, conventional asthma medications include bronchodilators and glucocorticoids. They focus on symptom control, are not curative, and are often ineffective in severe cases. Th cells remain at the forefront of asthma pathogenesis due to chronic airway inflammation and airway remodeling (**Figure 2**). Precision medicine targeting Th cells and related cytokines is essential in the management of asthma aiming at the more appropriate patient selection and better treatment response. This review focuses on Th cells and their cytokines in the pathogenesis of asthma and the associated targeted emerging therapeutic approaches.

## 2 “Type-2-high” asthma

### 2.1 Epithelial cytokines

Epithelial cytokines play a role as “whistle-blowers” in developing allergic responses at barrier surfaces and are involved in the pathogenesis of type 2 inflammatory diseases, typically asthma. The release of these “alarmins”, including IL-25, IL-33, and thymic stromal lymphopoietin (TSLP), occurs when the allergen stimulates bronchial epithelial cells (14). Although all of them are indispensable cytokines for initiating the Th2 immune response, they do not have the same role. IL-25 expresses at high levels in the lung of sensitized mice, promoting ASM contraction and increasing eosinophil recruitment and antigen-presenting ability (15, 16). In humans, levels of IL-25 receptors were increased in both myeloid cells and plasmacytoid dendritic cells (DCs) in the airways of patients with mild asthma after the allergen challenge (17). Bronchial epithelial IL-25 expression has a major role in triggering type 2 responses, with patients with higher IL-25 levels having greater AHR and more airway and blood eosinophils, higher serum IgE, more subepithelial thickening, and higher expression of Th2 signature genes (18). IL-33 induces signals in immune cells through its receptor IL-1RL1, which leads to the production of type 2 cytokines and chemokines (19). In mouse models, prophylactic or therapeutic blockade of IL-33 release attenuated asthma exacerbations (20). In allergic or eosinophilic asthma patients, IL-33 in bronchoalveolar lavage fluid (BALF) correlates with asthma severity (21). Interestingly, anti-inflammatory IL-37, whose target cells include airway epithelial cells (AECs), Th cells, and DCs, may limit the pro-inflammatory signaling of IL-33 (22). IL-37 reduced Th2 cytokine production in allergen-activated monocytes from wild-type but not IL-1R1-deficient mice and inhibited IL-33-induced Th2 cytokine release (22). In addition, IL-37 down-regulated allergic airway inflammation and attenuated IL-1β and IL-33-induced expression of pro-inflammatory mediators (22).

TSLP is considered to be a primary regulator of type 2 immune response to the respiratory barrier, which activates Th2 cells, innate lymphoid cells (ILCs)-2, mast cells, and other immune cells by inducing costimulatory molecules on DCs (23). Accumulated shreds of evidence are proposed to improve this view. Early studies have shown that compared with healthy controls, the expression of TSLP mRNA in the bronchial epithelium and submucosa of asthmatic patients is significantly increased (24). A large epidemiological study reveals that plasma high TSLP levels were associated with current asthma, poor lung function, and even the persistence of asthma attacks and dyspnoea 10 years later (25). Additionally, a part of patients with high TSLP expression shows hormone resistance indicating that glucocorticoid resistance asthma might be particularly relevant to the interaction of TSLP and ILCs (26). This is possibly due to ILCs expressing higher TSLPR decreasing the apoptosis of ILCs via the activation of STAT5 and the upregulation of (B-cell lymphoma-xl)Bcl-xl (26). In addition, TSLP also has convincing evidence for an effect on “Type-2-low” asthma. Human TSLP promotes the differentiation of Th17 cells with the central memory T cell phenotype through DCs activation (27). TSLP expression in human BALF was closely associated with neutrophil infiltration, and anti-TSLP therapy has been shown to reduce exacerbations in patients with severe, uncontrolled asthma with lower blood eosinophil and FeNO levels (28).

In addition, interactions between cytokines released by Th cells or other cells and AECs contribute to the progression of asthma. While higher levels of activin-A expressed by AECs may promote an increase in Th2 and Th9 cells (29), upregulating type 2 inflammation, IL-4 secreted by Th2 is involved in coordinating the polarization of AECs (30). Furthermore, there are epithelial indicators that are directly dependent on the cytokine profile of asthma patients and may play a part in the management of asthma (31). (The details will be described in turn below)

### 2.2 Th2 cells and type 2 cytokines

Th2-driven adaptive immune response and ILC2s-driven innate immune response are currently regarded as two independent but linked pathogenic mechanisms of asthma, both of which produce type 2 cytokines (32). ILC2s primarily respond to epithelial cell-derived “alarmins” with proliferation and activation (33, 34). ILC2s are unique in their high secretion of IL-9 can promote goblet cell metaplasia and promote mast cell growth and survival (35). Th2 cells, which produce IL-4, IL-5, and IL-13 in an antigen-dependent manner, are thought to play a major role in the pathogenesis of asthma (36). An array of those ‘type 2 cytokines’ exerts different and superimposed effects on the airway, which is pivotal to the pathological process of allergic asthma. IL-5 creates lung tissue and systemic eosinophilia, while IL-4 and IL-13 are involved in goblet cell metaplasia and AHR (37, 38). These changes in inflammation and remodeling further increase the susceptibility to exacerbation, thereby stimulating airway damage and subsequent remodeling (39). Besides, the interaction of Th2 cytokines and AECs may also be beneficial in maintaining a type 2 inflammatory environment. On the one hand, IL-4 antagonizes the IFN-γ-inducing genes expressed by AECs (30). On the other hand, IL-4 induces the expression of IL-24 in normal human AECs. IL-24 expression was elevated in nasal scrapings and sputum from patients with allergic rhinitis and seasonal asthma compared to healthy controls (40). IL-24 promotes airway remodeling by modulating epithelial–mesenchymal transition (EMT), and silencing IL-24 treatment attenuates airway inflammation, AHR, and airway remodeling in the asthmatic mouse model (41).

IL-5 (to a certain extent, IL-13 and granulocyte-macrophage colony-stimulating factor (GM-CSF)) controls eosinophils’ maturation, differentiation, and release (42). In the mouse model, IL-5 maintains the survival of lung-resident-like eosinophils, and IL-5 neutralization markedly reduces the percentage and the total number of lung and peripheral blood eosinophils (43). Also, sputum eosinophils and IL-5 were significantly elevated in asthmatic patients with accelerated decline in lung function (44). IL-5 promotes the proliferative properties of inflammatory-like and lung-resident-like eosinophils and the effect may be related to the expression of IL-5R in asthmatic patients (45). Eosinophils highly express IL-5Rα on their surface, and airway inflammation and injury occur once eosinophils are mobilized from the bone marrow and blood pool to the airway (46). Activated eosinophils are associated with endogenous AHR and airway inflammation with the release of reactive oxygen species and granulins, and interact with intraepithelial mast cells to regulate airway inflammation (47). Normally, eosinophils can survive *in vitro* for about 48 hours, however, IL-5 can prolong the survival time of eosinophils (48).

IL-4 and IL-13 share one type II receptor, directly or indirectly leading to various pathological changes of airway obstruction and AHR. IL-13 and IL-4 induce up-regulation of vascular cell adhesion molecule-1 (VCAM-1) expression on human endothelial cells and are involved in collagen production in fibroblasts (49). In addition, they enhanced the histamine-induced Ca^2+^ mobilization that was accompanied by increased mRNA expression of histamine H1 and cysteinyl leukotriene CysLT1 receptors, inducing glucocorticoid-insensitive AHR in isolated human small airways (50). Furthermore, more pieces of evidence confirmed that IL-13 mainly mediates physiological functions of airway remolding, including mucous cell metaplasia, smooth muscle cell contraction, goblet cell metaplasia and proliferation, and tissue fibrosis (51, 52).

### 2.3 T_FH_ cell

Accumulating emerging evidence suggests that T_FH_ cells, rather than Th2 cells, drive IgE responses in asthma. Using house dust mites (HDM) and mice, León and her colleagues found that after the first sensitizing exposure, T_FH_ cells reside in the draining mediastinal lymph nodes of the lungs and differentiate into Th2 and migrate to the lungs to exert their effects after a second attack by HDM allergens (53). Meanwhile, studies have also shown pulmonary cDCs effectively induced T_FH_ cells in asthmatic lungs and that the induced T_FH_ cells secreted IL-4 to support the production of IgE antibodies (54, 55). It is worth emphasizing that T_FH_ cells are not only necessary for IgE antibody production but are also sufficient even in the absence of typical Th2 cells or when their effector function is impaired (54). Further evidence suggests that a rare population of IL-13-producing T_FH_ cells is essential for the production of high-affinity IgE antibodies and subsequent allergen-induced allergic responses (9). In humans, the frequency of circulating CXCR5CD4T cells (T_FH_ cells) was elevated in allergic asthma, with increased production of IL-4, and IL-21, and positively correlated with total IgE in the blood (56). After responding to IL-4 and/or IL-13 from T_FH_ cells, memory IgG^+^ B cells begin to produce IgE (57). The interaction between IgE and FcϵRI on mast cells and basophils is an amplifier of the allergic cascade response, triggering increased vascular permeability and increased cellular recruitment, the release of mediators, lipid mediators, and cytokines (58).

### 2.4 Th9 cell

Th9 cells are a unique subpopulation of Th cells that mainly produce IL-9 as a signature cytokine. Increased numbers of Th9 cells were found in lung tissue, draining lymph nodes and airways of asthmatic mouse models, while anti-IL-9 antibody inhibits Th9 cell differentiation and attenuates allergic airway inflammation (59). Transcriptomic analysis revealed increased IL-9 expression in peripheral blood cells of patients with allergic eosinophilic asthma (60). IL-9 is mainly derived from Th9 cells (partly, ILC2 and Th2) and is a key factor in allergic airway inflammation. IL-9 promotes the pathogenesis of asthma by activating and recruiting mast cells and eosinophils, enhancing B-cell IgE production, increasing epithelial cell mucus production, and triggering airway AHR (29, 61). Natural allergen exposure raised local and systemic Th2, Th9, and reduced Tregs cells, but allergen-specific immunotherapy(AIT) inhibited Th2 and Th9 cell frequencies along with transforming growth factor (TGF)-β and IL-9 secretion (62). A crucial pro-inflammatory mediator in allergy reactions, activin A, a TGF-superfamily member connected to TGF-β1, also regulates Th9 development and effector activities (63). Musiol et al. found that targeting the TGF-β pathway may not only inhibit airway remodeling processes but also target pro-inflammatory Th2 and Th9 cells without adversely affecting immune tolerance in the asthmatic model (62). Another study found that activin-A was able to replicate the ability of TGF-β1 to drive Th9 polarization *in vitro* and *in vivo*. Crucially, effective inhibition of Th9 function *in vivo* is entirely dependent on the blockade of TGF-β and activin-A (29).

## 3 Biologicals targeting “Type-2-high” asthma

### 3.1 Biologicals targeting epithelial cytokines

Targeting these epithelial cytokines and associated signal pathways is considered an effective therapeutic strategy for asthma. Tezepelumab is a human monoclonal antibody that specifically binds to TSLP and blocks its interaction with the receptor complex, and is approved for severe asthma without phenotypic (e.g., eosinophilic or allergic) or biomarker restrictions. In phase 2 and 3 clinical trials, including patients with severe asthma, Tezepelumab (subcutaneous injection) consistently and significantly reduced acute asthma exacerbations regardless of key biomarkers (64, 65). In addition to this, the anti-TSLP treatment improved lung function, reduced airway eosinophil inflammation, and decreased the proportion of patients with AHR in asthmatic patients (66, 67). More in-depth studies on Tezepelumab to improve airway remodeling are possible in the future. Ecleralimab (CSJ117), a novel anti-TSLP monoclonal antibody fragment administered by inhalation, recently showed results from a phase I randomized controlled trial in mild asthma. After 14 weeks of administration (4 mg once daily inhaled), Eculalizumab was shown to be effective in attenuating allergen-induced responses in asthma patients as it significantly attenuated allergen-induced bronchoconstriction and airway inflammation (68).

Clinical trials with anti-IL-25 antibodies have not been conducted, but anti-IL-33 has obtained prospective results in a few trials. Itepekimab, a monoclonal antibody targeting IL-33, causes a reduction in blood eosinophils with a long-lasting effect in patients with moderate asthma (69). Results from another phase 2 trial showed that blocking IL-33 with itepekimab resulted in a lower incidence of uncontrolled asthma events compared to placebo, which was close to dupleuximab (22% vs 19%) and improved lung function (70). Astegolimab, a selective inhibitor of the IL-33 receptor ST2, was shown to attenuate the asthma exacerbation rate at a subcutaneous dose of 70 mg in a phase 2 trial in patients with severe asthma, including those with low eosinophils (71).

### 3.2 Biologicals targeting Type 2 Cytokines

#### 3.2.1 Biologicals targeting IL-5

Based on accumulating evidence implicating IL-4, IL-5, and IL-13 are responsible for the occurrence and development of asthma. Several clinical trials targeting type 2 cytokines are currently underway, and some of the relevant biologics have been approved for the treatment of severe asthma. Mepolizumab, Reslizumab (anti-IL-5 mAb), and Benralizumab (anti-IL-5R mAb) were FDA-approved to treat patients with severe eosinophilic asthma (37). Several prospective clinical trials have shown that the use of mepolizumab (administered subcutaneously or intravenously) or reslizumab (administered intravenously) in patients with asthma not adequately controlled by inhaled corticosteroids or severe eosinophilic asthma significantly reduced asthma exacerbations and was associated with improvements in markers of asthma control with a favorable safety profile (72–75). In addition to this, in patients with oral corticosteroid-dependent asthma, the use of reslizumab resulted in fewer new systemic corticosteroid prescriptions and a lower systemic corticosteroid burden (76). Besides, the effectiveness of mepolizumab and reslizumab is consistent with clinical trial results under real-world settings, with significant reductions in exacerbations and daily maintenance of oral corticosteroid dose (77, 78). Another biologic agent on IL-5, benralizumab, a monoclonal antibody directed against the alpha subunit of the IL-5R, has also shown significant clinically relevant benefits in patients with severe asthma (79). In several phase 3 trials, patients with severe asthma who received subcutaneous benralizumab achieved clinical benefits in terms of oral glucocorticoid use, lung function, asthma control, symptoms, asthma-related quality of life, and rate of exacerbation (79, 80). The most frequent adverse events were nasopharyngitis, worsening asthma, and bronchitis (80).

#### 3.2.2 Biologicals targeting IL-4 and IL-13

Likewise, the clinical trials of targeted IL-4/IL-13 therapy are also continuously increasing, with mixed results. Concerning IL-13, two biological agents have been studied, Lebrikizumab and Tralokinumab. In 2016, two phase 3 clinical trials of lebrikizumab for asthma (Lavolta I and Lavolta II), Lavolta I enrolled 2,000 patients with severe asthma and showed that lebrikizumab significantly reduced acute asthma exacerbations and also improved lung function in patients, yet similar results were not obtained in Lavolta II (81). A phase 3 trial in uncontrolled adolescent asthma patients found that Lebrikizumab reduced the rate of asthma exacerbation in uncontrolled adolescents, yet this study was terminated prematurely (82). Some trials found that lebrikizumab treatment was associated with improved lung function, especially in patients with high periostin levels (83). Another biologic, tralokinumab, has also not performed satisfactorily in severe asthma. Although tralokinumab reduced the annualized asthma exacerbation rate in participants with severe asthma with baseline FeNO 37 ppb or higher in the phase 3 clinical trial (STRATOS 1), it did not in another (STRATOS 2) (83). Further, William and his colleagues disappointedly found that asthma exacerbation rate and inhaled corticosteroid dosage were not significantly different between tralokinumab and placebo with uncontrolled asthma (84). Anti-IL-4 biologic, Pascolizumab, has not achieved better results in clinical trials of asthma and further studies have been discontinued. Bispecific antibodies that can simultaneously bind IL-4 and IL-13 have also entered clinical trials. Dupilumab is the first fully human anti-IL-4 receptor α monoclonal antibody approved for asthma that blocks both IL-4 and IL-13 signaling (85). In moderate to severe uncontrolled asthma, the incidence of exacerbations was significantly lower in patients treated with dupilumab than in those receiving a placebo, as well as better lung function and asthma control (85). Dupilumab also improved FEV_1_ and asthma symptom control, with greater efficacy observed in patients with elevated type 2 inflammatory biomarkers at baseline (86). In addition to the control of asthma symptoms and improvement in lung function, a multicenter study demonstrated that dupilumab reduced the use of oral corticosteroids in patients with corticosteroid-dependent severe asthma (87). The long-term efficacy and safety of dupilumab are also noteworthy. Further improvements in exacerbation rate, asthma control and health-related quality of life, and progressive reductions in blood eosinophils and total serum IgE were observed in patients treated with dupilumab at treatment extensions up to 148 weeks (88).

#### 3.2.3 Biologicals targeting IgE

By targeting the Fc fragment of IgE, Omalizumab, an anti-IgE monoclonal antibody, reduces free serum IgE levels and inhibits the binding of IgE to its high-affinity receptors on mast cells and basophils for the treatment of asthma. Compared with the other antibodies, Omalizumab was the first biologic approved by the FDA for the treatment of asthma. A review that included 25 clinical trials showed that omalizumab (mostly, subcutaneous injection) was effective in reducing asthma exacerbations and hospitalizations (89). Evidence from real-world studies suggests that the treatment of omalizumab in patients with asthma improved exacerbation rates and reduced hospitalizations, regardless of biomarker status (90). Dosing of omalizumab is based on body weight and pre-treatment serum total IgE levels, and its immunomodulatory effects persist after discontinuation. Although IgE levels do not accurately predict treatment efficacy, they may help predict cases requiring re-treatment after discontinuation (91). Serum inflammation-protective collagen 4 (COL4A3) levels are elevated in adults and children with asthma and are associated with a more severe, exacerbated allergic asthma phenotype. A cohort study evaluating patients treated with omalizumab using an asthma control test identified COL4A3 as a novel biomarker for predicting response to anti-IgE therapy (31). In biomarker analysis, a subgroup of patients with higher FeNO, peripheral blood eosinophil count, and serum periostin had relatively better treatment response^s^ (92).

#### 3.2.4 Biologicals targeting IL-9

IL-9-targeted therapy may offer a new approach to treating patients with asthma, but clinical studies are not well underway. In two randomized phase 2a studies in asthmatic subjects, patients who received MEDI-528, a humanized anti-IL-9 monoclonal antibody, showed a slight improvement in symptoms without a change in lung function (93). Another clinical trial using MEDI-528 in patients with moderate to severe asthma also failed to show efficacy (94). Perhaps this is because the heterogeneity of asthma makes it difficult for MEDI-528-targeted therapy to show significant beneficial effects in non-selected asthma populations (94). Current biological agents in “Type-2 high” asthma are summarized in **Table 1**.

**Table 1:** Current biological agents in “Type-2 high” asthma.

Related cell	Therapeutic Target	Biologic Agent	FDA approved	Route of Administration and Dose	Efficacy
Epithelial cells	TSLP	Tezepelumab	Yes	SC; 210 mg every 4 wks	Reduced exacerbations and AHR; decreased eosinophilic inflammation; Improved lung function and quality of life
	TSLP	Ecleralimab	No	Inhal; 4mg qd	attenuated bronchoconstriction and airway inflammation
	IL-33	Itepekimab	No	SC; 300mg every 2 wks	Reduced exacerbations; decreased eosinophilic inflammation; improved lung function
	IL-33/ST2	Astegolimab	No	SC; 70-490mg every 4 wks	Reduced exacerbations
Th2 cell	IL-5	Mepolizumab	Yes	IV; 75mg every 4 wks SC;100mg every 4 wks	Reduced exacerbations; Improved quality of life, lung function, and markers of asthma control
	IL-5	Reslizumab	Yes	IV; 3.0mg/kg every 4 wks	Reduced exacerbations and daily maintenance OCS;
	IL-5R	Benralizumab	Yes	SC; 30mg every 4 wks	Reduced exacerbations and daily maintenance OCS; Improved lung function (in part study), and symptoms
	IL-4	Pascolizumab	No	N/A	N/A
	IL-13	lebrikizumab	No	SC; 37.5 mg or 125 mg every 4 wks	Unsatisfactory (Inconsistent across studies)
	IL-13	Tralokinumab	No	SC; 300 mg every 2 wks	Unsatisfactory (Inconsistent across studies)
	IL-4 and IL-13	Dupilumab	Yes	SC; 200 or 300mg every 2 wks	Reduced exacerbations; Improved lung function and asthma control; suppressed type 2 inflammatory biomarkers
T_FH_ cell	IgE	Omalizumab	Yes	SC; 75 to 375 mg every 2 to 4 wks (based on body weight and pretreatment level of serum total IgE)	Reduced exacerbations, improved quality of life, lung function, and asthma control
Th9 cell	IL-9	MEDI-528	No	SC; 30-300 mg every 2 wks	Unsatisfactory

## 4 “Type-2-low” Asthma

### 4.1 Th1 in “Type-2-low” asthma

Th1 inflammation is mainly characterized by an increase in Th1 cells and infiltration of signature cytokines including IFN-γ and TNF-α. Because of the inhibition Th2 inflammatory response, Th1 is considered to have an inhibitory effect on asthmatic airway inflammation in early investigations. Several studies have shown that IFN-γ inhibits Th2 cell function to attenuate the inflammatory response in asthma (95). Further, Mitchell et al. found that IFN-γ acting through the airway epithelium inhibits mucus, chitinases, and eosinophilia, independent of Th2 cell activation (96).

Growing amounts of data are showing a link between Th1 cells and the emergence of “Type-2-low” corticosteroid-resistant asthma. AHR, neutrophilia, and airway remodeling were persistent in mice with elevated Th1 cells during allergic airway inflammation, and these mice were resistant to corticosteroids (97, 98). Wisniewski and colleagues discovered that IFN-γ^+^CD4^+^ T cells were concentrated in the BALF of adult patients with severe asthma and that Th1 airway inflammation was evident in children with allergic and non-allergic severe asthma (11, 99). ASM is an important target for corticosteroid therapy and IFN-γ and TNF-α have a significant effect on ASM, which is responsible for the insensitivity to corticosteroids in patients with severe asthma. Rodney et al. used human fetal ASM as an *in vitro* model and found that the interaction between TNF-α and IFN-γ mediated pathways may promote corticosteroid resistance during ASM development (100). In a mouse model, inhibition of TNF-α resulted in a decrease in airway remodeling-related mediators (101). Raundhal et al. revealed that IFN-γ levels are inversely correlated with secretory leukocyte protease inhibitors (SLPI) expressed by airway epithelial cells, dysregulation of the IFN-γ/SLPI axis involves the AHR and corticosteroid refractoriness (11). The transcriptomic analysis also revealed that combined exposure to TNF-α and IFN-γ affects several gene expressions, which impairs corticosteroid sensitivity in ASM and may lead to persistent symptoms and worsening of severe pediatric asthma (102). Recently, several interesting studies have found that obesity-associated asthma patients are associated with Th1 activation, neutrophil inflammation, and pulmonary function deficits (103). Th cell transcriptome analysis identified upregulation of genes in the cytokinesis cycle 42 pathway (associated with TNF-α and IFN-γ expression) in Th cells of children with obesity-related asthma (5).

### 4.2 Th17 in “Type-2-low” asthma

Current researches reveal that Th17 cells and their cytokines are linked to some, but not all, “Type-2-low” asthma, particularly in severe asthma with neutrophil predominance.

Many well-known cytokines work together for Th17 cell differentiation to occur, with IL-23, IL-6, and TGF-βbeing the main drivers of this process. IL-6 in combination with TGF-β induced differentiation of naïve T cells to Th17 cells, which is the cornerstone in autoimmune diseases (104). Activated Th17 cells release more IL-6 to further promote Th17 cell differentiation (104). IL-23, a member of the IL-12 family of cytokines, is released primarily by DCs, macrophages, B cells, and endothelial cells and is essential for the survival and effector capacity of Th17 cells (105). In the absence of IL-23, Th17 cells stimulated by TGF-β and IL-6 showed impaired pathogenic effects despite increased IL-17 production (106). IL-23 enhances eosinophil inflammation in “Type-2-high” asthma. Inhaled allergen stimulation in asthmatic mice elevated IL-23 expression in the bronchial epithelium and eosinophil infiltration, whereas IL-23 inhibition reduced airway inflammation and hyperresponsiveness (107). Moreover, IL-23 plays an essential part in neutrophil recruitment. According to Wakashin et al., forced expression of IL-23 in sensitized mice’s airways increased the production of IL-17A in response to antigens and the recruitment of neutrophils (108). In comparison to healthy populations, IL-23 activation of peripheral blood neutrophils isolated from asthmatic patients dramatically boosted IL-17A and IL-17F production (109).

Th17 cells secrete IL-17A, IL-17F, IL-21, IL-22, and TNF-α to perform their functions. IL-17A and IL-17F are believed to contribute to asthmatic neutrophil inflammation. Experimental mouse models of asthma suggest that IL-17A enhances the recruitment of bronchial neutrophils through CXCR2 signaling (110). Serum levels of IL-17A were also higher in asthmatic mice than in the wild type, implying that IL-17A may also promote neutrophil transmigration (111). Sorbello et al. found increased expression of IL-17F and IL-17A in the nasal and bronchial lamina propria and higher expression of bronchial neutrophils in patients with severe asthma (112). An immunohistochemical study of bronchial and nasal biopsies found that bronchial and nasal IL-17F correlated with bronchial neutrophil counts, asthma exacerbation rates, and FEV_1_ (113). Bullone et al. conducted an interesting study in an attempt to characterize the immunopathology of neutrophilic asthma and to identify potential clinical markers for this subtype. They performed bronchial biopsies on 70 asthmatic patients and identified the “high neutrophil” subtype. The final results showed a significant increase in IL-17F-positive cells in patients with high neutrophils. After excluding smokers, an increase in IL-17A was also found in highly neutrophilic patients (114). It has also been found that IL-17A and IL-17F exert different biological effects on the airway inflammation of asthma. Several studies have associated IL-17A with airway remodeling and AHR, the major physiological characteristics of asthma. IL-17A was found to induce mucus cell chemotaxis independent of STAT6 by establishing a mouse model (115). A study quantifying bronchial angiogenesis as well as the number of IL-17A cells and the concentration of angiogenic factors in sputum in subjects with severe asthma, COPD, and healthy subjects found that IL-17A enhances bronchial vascular remodeling by stimulating the synthesis of other angiogenic factors may lead to mucosal congestion resulting in airway narrowing (116). Other studies have found that IL-17F, but not IL-17A, underlies airway inflammation in steroid-insensitive toluene diisocyanate-induced asthma models and that anti-IL-17F ameliorates toluene diisocyanate-induced AHR and airway neutrophilia with a reduced Th17 response (117).

Other cytokines, IL-21 activate Th17 in an autocrine manner (118), while TNF-α is associated with airway inflammation in severe asthma. Patients with neutrophilic asthma were shown to have mucosa-associated invariant T cells that were predisposed to the Th17 subtype, which released higher levels of IL-17A and TNF-α and lower levels of IFN-γ (119). Niessen et al. found that sputum TNF signaling protein was more significantly elevated in neutrophilic asthma and that elevated sputum TNF signaling protein in patients with severe asthma was associated with poorer lung function and worse asthma control (120). In addition, TNF-α in combination with IL-17 altered the bronchial epithelial cell proteome to enhance proteins that promote neutrophil migration (121).

Th17-related cytokines are also relevant to obesity-associated asthma. Late-onset asthma patients with typical features of obesity-associated asthma are mostly female and involve neutrophilic airway inflammation and oxidative stress (122). In mice with obesity-associated asthma, TNF-α increases metalloproteinase (MMP)-9 and *in situ* gelatinase activity and contributes to airway remodeling (123). According to multiple research, children, and adults with obesity-associated asthma had higher peripheral blood levels of IL-17A, IL-21, and TNF-α than non-obese patients (124, 125).

### 4.3 Th22 in “Type-2-low” asthma

IL-22 is produced by Th22, but this cytokine can also be secreted by other cells including Th1, Th2, and Th17 (13). Similar to the results in mice, the study found elevated serum and sputum IL-22 levels in asthma patients compared to healthy samples suggesting that IL-22 may be involved in the pathogenesis of asthma (126, 127). Although some studies suggest that IL-22 may exhibit inhibitory effects on airway inflammation in asthma by inhibiting the action of IFN-γ on epithelial cells (13), there are still few studies exploring the mechanisms of IL-22 and human asthma pathophysiology.

## 5 Biologicals targeting “Type-2-low” asthma

### 5.1 Biologicals targeting TNF-α

TNF-α, which is released by Th1 cells, Th17 cells, mast cells, and macrophages, is thought to be an attractive target in severe asthma. A case report identified that asthma symptoms may be masked by anti-TNF-α therapy, suggesting that anti-TNF-α may have a therapeutic effect on asthma (128). The recombinant protein Etanercept (ETN), which binds TNF with a high affinity, is made up of two molecules of the extracellular region of the p75 TNF-α receptor. In the Holgate et al. randomized controlled study, patients with asthma showed no discernible changes in treatment objectives between ETN and placebo (129). Another study found that administration of ETN for 12 weeks modestly reduced AHR, improved asthma symptom control, quality of life, and lung function, and reduced the expression levels of membrane-bound TNF on peripheral blood mononuclear cells (130). Other anti-TNF-α treatments, such as infliximab, adalimumab, and golimumab, have similarly failed to provide promising outcomes (131). Although anti-TNF-α has been shown to improve asthma control in animal models, patients with severe asthma have not benefited from this therapy because there is limited evidence of improved lung function and reduced asthma exacerbation. More studies are needed to further explore it.

### 5.2 Biologicals targeting IL-6 and IL-23

IL-6 can induce Th17 differentiation, and blocking IL-6 may be a potential target for the treatment of asthma. Tocilizumab, a humanized anti-IL-6 receptor (IL-6R) mAb, was used in two patients with recurrent acute exacerbations of asthma, both of whom showed a good clinical response and reduced hospital admissions (132). Clazakizumab, another anti-IL-6 monoclonal antibody, is part of the PrecISE clinical trial, which uses a biomarker-guided approach to identify patient groups most likely to respond to one of six predefined interventions (133). Clazakizumab’s target population is defined as a subgroup characterized by obesity and metabolic dysfunction. Other clinical trials on targeted IL-6 interventions for asthma are not currently well underway, and a phase 2a study evaluating the impact of Sirukumab in subjects with poorly controlled severe asthma was terminated in early may due to safety concerns (NCT02794519). Another study evaluating the efficacy and safety of FB704A in adults with severe asthma is still enrolling patients with uncertain results (NCT05018299).

IL-23 is a pro-inflammatory cytokine regulated in Th17 and targeting IL-23 may be particularly beneficial in neutrophilic asthma. In a randomized, double-blind, placebo-controlled phase 2a study, IL-23 p19 inhibitor, Risankizumab was used in adult patients with severe asthma. However, disappointingly the study did not meet the primary endpoint. time to first asthma worsening was shorter in the risankizumab group than in the placebo group, and the annualized rate of asthma worsening was higher in the risankizumab group than in the placebo group. Lung function, ACQ-5 scores, and the incidence of severe asthma exacerbations were similar in both groups (134).

### 5.3 Biologicals targeting IL-17

Targeting IL-17 in neutrophilic asthma is also being studied continuously. Brodalumab is an anti-IL-17 receptor monoclonal antibody for which Busse et al. conducted a placebo-controlled phase 2a clinical trial for the treatment of moderate-to-severe asthma. Subjects requested discontinuation of a long-acting beta-agonist (LABA) for the duration of the study, and inhaled glucocorticoids were available. At week 12, Brodalumab did not produce any statistically significant benefit in terms of ACQ scores, FEV_1_, or short-acting β2-agonist (SABA) use (135). BITS7201A is a novel humanized bispecific antibody that binds and neutralizes IL-13 and IL-17. A phase I trial investigating the effect of BITS7201A on asthma was terminated early due to high antibodies to therapeutic agents (136). Secukinumab, a fully human anti-IL-17A monoclonal antibody, is now used in rheumatic immune diseases, and a clinical trial has attempted to evaluate its role in patients with asthma not adequately controlled with inhaled corticosteroids but has failed to obtain satisfactory results (NCT01478360). Badi et al. found that the degree of enrichment of similar transcriptomic features in atopic dermatitis, including overexpression of Th17 and Th22-related genes, was strongly associated with severe neutrophilic asthma. Then, patients with severe neutrophilic asthma were identified as potential responders to Fezakinumab (anti-IL-22, currently approved for atopic dermatitis treatment) therapy (137). Although some trials have reported different data against Th17-related cytokines, the safety, and efficacy remain unsatisfactory. Perhaps the selection of a more appropriate “type-2 low” asthma patient for precisely targeted therapy is the key to successful trial progress. Current biological agents in “Type-2 low” asthma are summarized in **Table 2**.

**Table 2:** Current biological agents in “Type-2 low” asthma.

Related cell	Therapeutic Target	Biologic Agent	FDA approved	Route of Administration and Dose	Efficacy
Th17	IL-6	Tocilizumab	No	IV; 8-10mg/kg every 4wks	Reduced neutrophilic inflammation, improved lung function, and asthma control (2 cases)
	IL-6	Sirukumab	No	SC;50mg every 4wks	N/A (withdraw)
	IL-6	Clazakizumab	No	N/A	N/A
	IL-6	FB704A	No	N/A	N/A (recruiting)
	IL-23	Risankizumab	No	IV; 90mg every 4wks	Unsatisfactory
	TNF-α	Etanercept	No	IV; 25mg every 2wks	Unsatisfactory
	IL-17	Secukinumab	No	N/A	Unsatisfactory(terminated)
	IL-17RA	Brodalumab	No	SC; 140-280 mg at day 1 and wks 1, 2, 4, 6, 8, and 10	Unsatisfactory
	IL-13 and IL-17	BITS7201A	No	IV;150-600mg every 4wks	Unsatisfactory
Epithelial cells	TSLP	Tezepelumab	Yes	SC; 210 mg every 4 wks	Reduced exacerbations and AHR; decreased eosinophilic inflammation; Improved lung function and quality of life
	IL-33/ST2	Astegolimab	No	SC; 70-490mg every 4 wks	Reduced exacerbations

N/A, not applicable.

### 5.4 Treatment of asthma

**Figure 3:**
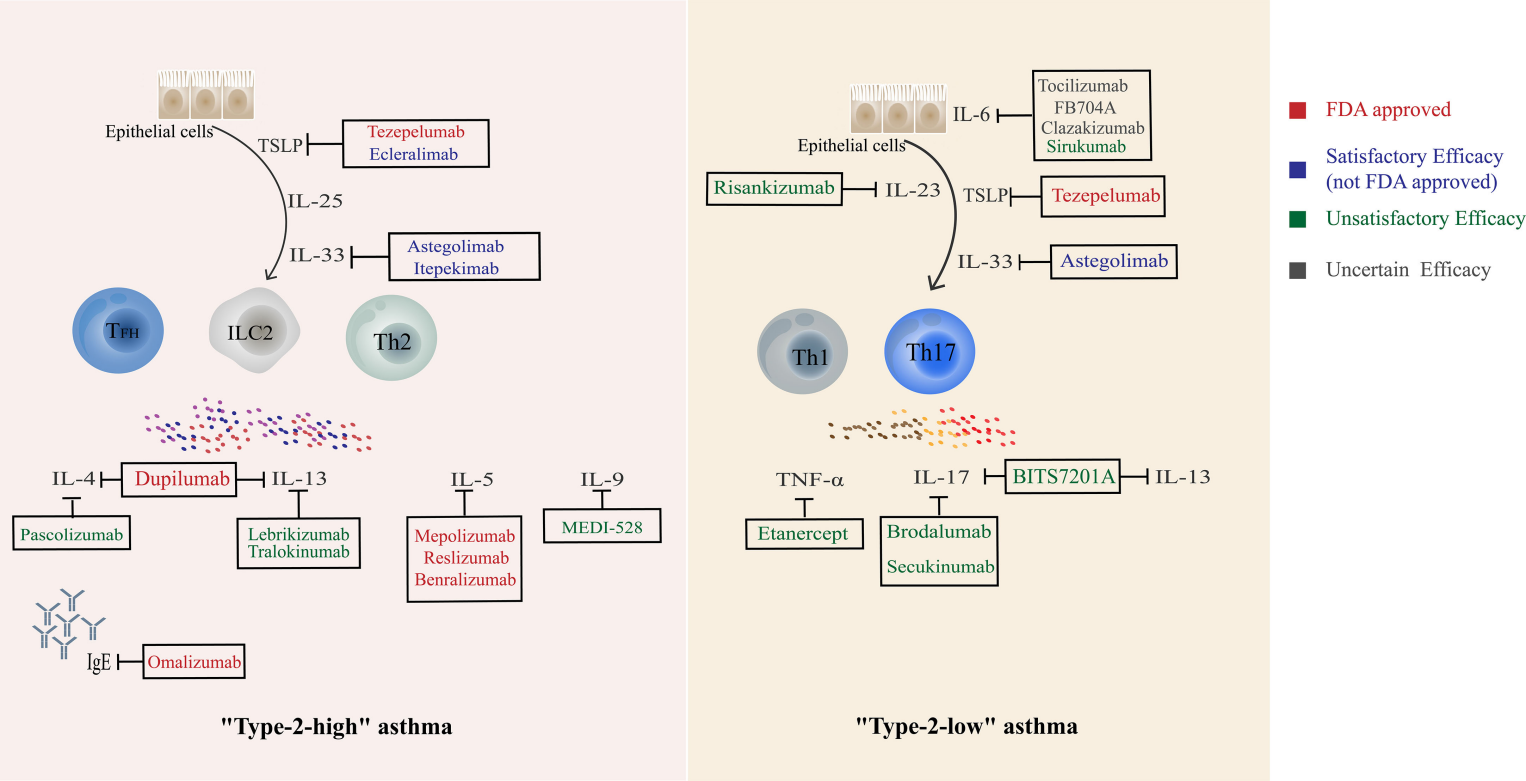
Current biological agents targeting Th cells and related cytokines.

The current focus of asthma treatment remains on pharmacotherapy, and the main drugs can be divided into bronchodilators such as SABA which provide rapid symptomatic relief by relaxing ASM and control drugs that inhibit inflammation represented by inhaled corticosteroids (ICS) (138). In the confrontation of severe asthma, additional biologic therapies can significantly reduce severe acute exacerbations and ICS exposure (138). In addition, other types 2 coexisting conditions (allergic rhinitis, eczema/atopic dermatitis, urticaria, etc.) are associated with a higher risk of exacerbations and lower asthma control, and clinicians may consider assessing the burden of type 2 comorbidities when evaluating patients with asthma (139). Th cells immune response plays an important role in the initiation and persistence of asthma, and biological agents targeting its cytokines and pathways are constantly updated (**Figure 3**). The vast majority of FDA-approved biologics (including benralizumab, dupilumab, mepolizumab, et al) are for “Type-2 high” asthma, with indications for severe eosinophilic asthma and/or oral corticosteroid-dependent asthma. Typical eligibility criteria for each category, as well as predictors of a good response, can be found in the GINA pocket guide decision tree (140). In the ALLIANCE cohort, patients in all age groups exhibited a T2-high phenotype characterized by eosinophilia and atopy, with the highest prevalence of T2-high asthma in school-aged children and young adults. Using easily accessible biomarkers, patients with T2-high asthma can be identified across all ages delineating a distinct phenotype. These patients may benefit from therapy with biologicals even at a younger age (141). Some other biologics with similar targets have achieved satisfactory clinical results and are progressing to the next stage of the study. In contrast, the progress of biologics for “Type-2 low “ asthma has been bumpy. Tezepelumab, the only indication for severe asthma (not just eosinophilic asthma), was found to be also effective regardless of key biomarkers (67). Astegolimab did a similar effect in clinical trials (71). Other biologics targeting Th1 or Th17 have shown poor results in several clinical trials. It is known that “Th1, Th17” may play essential roles in “Type-2-low “ asthma, and a more in-depth study of the pathological mechanism and the selection of the appropriate patient population based on the corresponding clinical indicators may bring about a turnaround. Larger studies are needed to investigate which asthma phenotypes benefit most from biologics that target Th17 and Th1-related cytokines.

AIT is the only disease-modifying and potentially preventive asthma treatment based on repeated injections or sublingual administration of specific allergens to allergic individuals to induce immune tolerance (142). The mechanism of AIT is primarily based on Treg cells, which downregulate allergic inflammation by producing the cytokines IL-10 and TGF-β (142). AIT slows local airway inflammation and inhibit pro-inflammatory CXCL8, IL24, and CCL26 mRNA expression (143). In addition, chronic allergen exposure resulted in high expression of CTLA-4 and PD-1 in Th2 cells and the prolonged presence of pro-allergic Th2 cells with a depleted phenotype during AIT may explain the long-lasting effect of AIT treatment (144). Several biomarkers were identified that may predict the clinical efficacy of AIT, such as the production of allergen-specific IgG or IgG4 and IgA in serum, suppression of local Th2/ILC2 cell numbers, or secretion of type 2 cytokines, increase in Th1 cell, Treg or Bregs numbers, and induction of a recently introduced anti-inflammatory mediator, secretoglobin 1A1, in the local environment (143, 145). An interesting study hypothesized three consecutive phases of AIT, namely an initiation, a conversion, and a tolerance mounting phase. In this study, the ratio of IL-10 B-cells and Th17 cells during the early initiation phase corresponded to symptom improvement after three years of treatment, representing a potential decision point for treatment adjustment before long-term treatment (146). AIT as an evolving area of asthma treatment still needs to be better understood.

## 6 Discussion

Complex interactions between cellular participants and the innate and adaptive immune systems in response to multiple allergenic stimuli coordinate the immune response in the lung. In Type-2 high asthma, T_FH_ cells control IgE synthesis by secreting IL-4 to allergen-specific B cells. Epithelial-derived TLPS, IL-25, and IL-33 can awaken ILC2 and Th2, while IL-4, IL-5, and IL-13 play a central role in the subsequent series of transformations. In the past few years, significant advances in Th17 and Th1 cell research have revealed new aspects of the “Type-2 low” asthma cellular machinery. Th1 and Th17-related cytokines act as central effector mediators and continuity factors in airway inflammatory storms and airway injury.

At present, the efficacy and safety of various biologics for Th2 responses have been well evaluated. Several biologics have been approved by the FDA for the treatment of “Type 2 high” asthma, such as benralizumab, dupilumab, and mepolizumab. They have demonstrated favorable results in clinical trials and real-world studies on reducing exacerbations, improving quality of life and lung function in patients with severe asthma, and even reducing OCS doses. However, various biologics for Th1 and Th17 are still struggling to move forward. Two biological agents (Tezepelumab and Astegolimab) have shown partial effects on “Type-2 low” asthma by blocking epithelial cell cytokines. More in-depth study of the pathological mechanism and the selection of the appropriate patient population based on the corresponding clinical indicators may bring about a turnaround. There is no silver bullet in asthma treatment. Asthma treatment may be individualized in the future. Perhaps more in-depth research on Th cells and big data analysis to match different clinical manifestations of asthma with specific Th cells for targeted and individualized treatment will be the new direction in the future.

## Author contributions

TJ, access to literatures and writing of the manuscript. HL, revising the manuscript. Both authors contributed to the article and approved the submitted version.
